# How does mindfulness training affect attention and penalty kick performance in university football player

**DOI:** 10.1371/journal.pone.0327134

**Published:** 2025-06-25

**Authors:** Jiaqi Wu, Lixin Ai

**Affiliations:** 1 College of Sports Science, Tianjin Normal University, Tianjin, China; 2 Department of Physical Education, Tianjin University of Finance and Economics Pearl River College, Tianjin, China; Ordu University, TÜRKIYE

## Abstract

Athletes often struggle to maintain attentional focus and performance consistency under pressure, particularly during high-stakes tasks like penalty kicks. This study examined the effects of brief mindfulness training on visual attention behaviors and penalty kick performance among university football players under non-pressure and pressure conditions. The study comprises two experiments: Experiment 1 was conducted in non-pressure conditions, whereas Experiment 2 involved pressure condition. Each experiment involved 40 participants, who were randomly assigned to either the intervention group or the control group. The intervention group underwent 15 minutes of brief mindfulness training, while the control group engaged in 15 minutes of quiet sitting. Eye-tracking technology was used to measure fixation count and fixation duration during the penalty kick tasks. In non-stress conditions, the mindfulness group showed a significant increase in fixation duration and fixation count, with no change in penalty kick performance. In pressure conditions, the mindfulness group again showed significant gains in fixation duration and fixation count, but penalty kick scores did not significantly improve. Comparisons across stress conditions revealed that fixation duration improvements were larger under pressure, suggesting mindfulness may enhance attentional stability when athletes are under stress. In conclusion, brief mindfulness training can enhance visual attention behaviors in university football players, especially under pressure, by promoting sustained focus on task-relevant visual targets.

## Introduction

Maintaining focus and consistent performance under high-pressure conditions is critical for athletes, particularly during penalty kick tasks in football. Penalty kicks not only require exceptional technical proficiency but also demand that athletes remain composed and focused in the face of psychological pressures from live audiences, opponent interference, and the intense atmosphere of competitive matches. Anxiety and stress often disrupt a player’s attention and decision-making clarity, thereby negatively affecting their penalty kick performance [1]. In this context, attentional control is one of the key factors influencing athletic performance [2], especially in precision-dependent tasks such as penalty kicks. Compared to self-report questionnaires and behavioral paradigms, eye-tracking offers a more direct and objective method for assessing attention [3,4].Previous research has consistently demonstrated that fixation behaviors, such as average fixation duration and fixation count, are more pronounced in task-relevant areas compared to task-irrelevant ones, reflecting stronger goal-directed attentional control [3,5]^.^ Visual search, characterized by the coordination of fixations and saccades, is a complex cognitive process that allows athletes to rapidly acquire task-relevant information in dynamic sports environments [6]. Research has shown that effective visual search strategies, particularly longer fixation durations on task-relevant areas, are associated with superior decision-making and motor execution across various sports such as basketball, fencing, darts, and billiards [7–10]^.^ These findings support the use of eye-tracking not only as a tool for measuring overt visual attention but also as an indicator of cognitive preparation and motor readiness. Specifically, longer fixations have been linked to improved performance by allowing athletes to better encode relevant information, reduce distraction, and optimize movement execution. Thus, fixation duration and search patterns can serve as meaningful indices of attentional control and performance quality in sport contexts.

Mindfulness training, defined as the practice of maintaining non-judgmental awareness of present-moment experiences [11], has gained attention as a strategy to enhance attentional control and emotional regulation in sports. Long-term mindfulness interventions have shown promising results in improving athletes’ emotional stability and performance consistency [12–14]. However, the feasibility of long-term programs is limited for athletes with tight schedules, prompting interest in brief mindfulness interventions. Although meta-analyses suggest that mindfulness can enhance attentional control and reduce anxiety under pressure [15], evidence on the effectiveness of brief interventions is mixed. Some studies report positive cognitive and emotional benefits [16,17], while others show no improvement or even detrimental effects on performance [18] Moreover, individual differences such as personality traits and intervention adherence appear to moderate these effects [19]

Despite these inconsistencies, brief mindfulness training remains an attractive approach due to its practicality. To date, some studies have examined the effects of brief mindfulness training on athletes’ psychological and athletic performance, such as improving attentional control in shooters [16], enhancing free-throw performance in basketball players [20], mitigating ego depletion [21], improving golf putting performance, flow experiences, and reducing state anxiety [22], and regulating cognitive functions in athletes [23]. However, the impact on football players’ performance and attentional control has yet to be validated. Penalty kicks involve high-pressure, precision-based actions that rely on attentional control and emotional regulation. Given football’ s global popularity and the decisive nature of penalty kicks, the findings may also apply to other high-pressure performance tasks. Therefore, we aim to investigate the effects of brief mindfulness training on football players’ performance and attentional control. Additionally, to better understand the true effects of brief mindfulness training, we conducted an experiment with another group of participants under pressure conditions. This approach allows us to explore the impact of brief mindfulness training on football players’ performance and attentional control under pressure, thereby examining the differences in the effects of brief mindfulness training on attention and performance in football players under different pressure conditions. To ensure the internal validity of the design and avoid potential confounds, the two experiments were conducted using independent participant samples. This approach was chosen to eliminate carryover effects, emotional residue, and potential learning or expectation biases that could arise if the same participants were subjected to both non-pressure and pressure conditions. While both experiments recruited university football players from the same population, they were independently assigned to either the non-pressure or pressure study arms to maintain experimental control.

**Fig 1:**
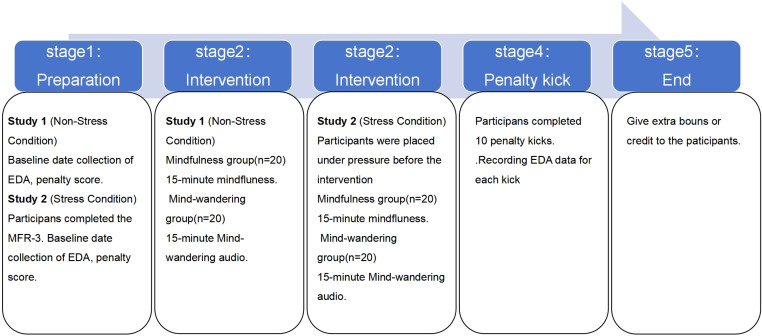
Flowchart of experimental procedures.

Thus, the purpose of this study is to explore the effects of brief mindfulness training on football players’ performance and attentional control under varying levels of pressure. Both intervention groups in the two studies underwent 15 minutes of brief mindfulness training, while the control group engaged in 15 minutes of quiet sitting. In Study 1, participants were under non-pressure conditions, whereas in Study 2, participants were subjected to pressure conditions. Based on previous research findings, we hypothesize that: (1) participants who receive brief mindfulness training will demonstrate longer fixation durations than the control group, both under non-pressure and pressure conditions [24,25]; (2) participants who receive brief mindfulness training will exhibit a greater number of fixations than the control group, both under non-pressure and pressure conditions [9,10]; (3) Participants who receive brief mindfulness training will achieve higher penalty kick scores than the control group, both under non-pressure and pressure conditions [20,24]; (4) the intervention effects of the mindfulness group may be more effective under pressure conditions, leading to higher football accuracy, longer fixation duration, and a greater number of fixations [15,26]. The flowchart of experimental procedures is shown in Fig 1.

## Study 1: Mindfulness training affect attention and penalty kick performance—Non-stress condition

### Participants

Based on a priori power analysis using G*Power version 3.1.9.2, targeting a 2 (Group: mindfulness vs. control) × 2 (Time: pre vs. post) mixed ANOVA, a minimum sample size of 34 participants was required to detect a medium effect size (f = 0.25) with α = 0.05 and power (1-*β*) = 0.80 [15]. To account for potential attrition, 42 male university football players were recruited for Study 1(The start date of the recruitment period for this study is September 26, 2024 and the end date is November 31, 2024). All participants were male university-level athletes, right-footed, had at least three years of football experience, no prior mindfulness training, and had not experienced any major life events in the past three months. Participants were required to have normal or corrected-to-normal vision (e.g., using glasses or contact lenses if needed) and no history of visual impairments such as strabismus, amblyopia, or uncorrected refractive errors that could interfere with eye-tracking calibration and data quality. Participants were recruited through team briefings and informational posters within the university football program. Participation in the study was voluntary, and participants were informed that the research aimed to investigate factors affecting attention and football performance, without disclosing the specific focus on mindfulness training to reduce expectancy effects. Participation in the study was voluntary, and as part of the single-blind design, participants were unaware of the study’s purpose. Cluster randomization was used to assign participants to either the control group or the experimental group.

In Study 1, valid data were obtained from 42 participants, with one excluded due to strabismus and another for not completing the study. The remaining 40 eligible participants were randomly assigned to the MM group (n = 20) or the MW group (n = 20). The average age of the participants was 20.7 ± 2.41 years old. All participants were male, right-footed university football players, experience over a 3–5 year period. This study was approved by the Ethics Committee of Tianjin Normal University (No: 2024092501), and informed consent was obtained from all participants prior to the start of the study.

### Procedures

During the preparation phase, each participant was individually tested by the experimenter. Participants were first introduced to the tasks and signed informed consent forms. They were then fitted with an eye-tracking device and familiarized themselves with its use. Participants completed a penalty kick test. Participants in the mindfulness group (MM group) received a 15-minute guided mindfulness breathing session using the “Yangqi” app (https://apps.apple.com/cn/app/id1509603275?l=ca), which incorporated breathing awareness, body scanning, and non-judgmental attention exercises. Participants listened to audio-guided instructions via headphones, helping to maintain engagement and ensure adherence throughout the session.

Participants in the MW group were asked to sit quietly in a chair for 15 minutes, which involved no structured cognitive exercises or mental training. At the end of the study, participants ranking in the top three received cash rewards of 50, 30, and 20 RMB, respectively, while others were compensated with extra course credit. Participants were informed about the reward system only after completing the pre-test measurements to minimize expectation effects.

## Measurements

### Penalty kick experiment

**Fig 2:**
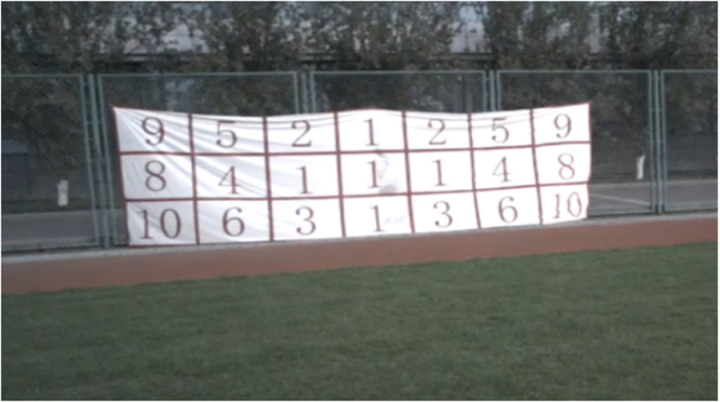
The distribution of goal scores in the penalty task.

The goal area was divided into 21 numbered regions, as shown in Fig 2, with larger numbers representing higher scores. Specifically, zones labeled 9 and 10, located near the corners and edges, were assigned the highest scores, reflecting higher difficulty and greater precision requirements. Zones with lower numbers (1–6) represented easier central target areas with lower associated scores. Participants’ penalty kick performance was measured by summing the numerical values of the zones they hit across 10 consecutive penalty kicks (Fig 2). This scoring system was adapted from the standardized goal area division used in the football specialty examination at Beijing Sport University. The division is based on the difficulty level of scoring from different areas of the goal, with higher points assigned to shots placed in more challenging areas such as the corners. This approach allows for a more nuanced quantification of penalty kick performance, moving beyond the binary outcome of ‘goal’ or ‘no goal’. By providing a continuous performance scale, it facilitates more detailed analysis of kicking accuracy and its relationship with eye-tracking data. In Study 2, participants were additionally instructed to complete the process—from placing the ball to kicking it—within 3 seconds to simulate a time-constrained, high-pressure situation, and no time limit was imposed in the no-pressure condition in Study 1.

### Eye-tracking hardware

The study utilized the Tobii Pro Glasses 3 portable eye tracker to collect data on fixation duration and fixation count during the penalty kick task. The eye tracker established a local area network connection with the main experimenter’s device to monitor participants’ eye movement metrics. Participants wore the eye tracker throughout the experiment, and data recording commenced after calibration. The device was set to a sampling frequency of 100 Hz to ensure the capture of detailed fixation behavior. The Tobii Pro Glasses 3 is equipped with four independent eye-tracking cameras, offering a precision of approximately 0.5°and an accuracy of around 0.1°. The device provides high-precision eye movement tracking in participants’ everyday activities, with a field of view of 120°horizontally and 90° vertically, allowing for the capture of a broad visual field.

### Statistical analyses

First, the collected data were subjected to preliminary preparation, which included ensuring data completeness and accuracy, followed by outlier screening and normality testing. Descriptive statistics were conducted for baseline measurements, and homogeneity was tested using independent samples t-tests. Normality was assessed using the Shapiro-Wilk test, and sphericity was evaluated using Mauchly’s test, both of which confirmed the data met analysis assumptions. The effects of brief mindfulness training on mindfulness levels, penalty kick performance, and eye-tracking data were examined using a repeated measures ANOVA, with the intervention group and control group as between-subject factors, and pre- and post-intervention time points as within-subject factors. A 2 × 2 matrix was employed, comparing the experimental group with the control group, and pre-intervention measurements with post-intervention measurements. In this framework, the “group” variable served as the between-subject factor, while the “time” variable acted as the within-subject factor. A key part of the analysis was the interaction between “time” and “group.” Detecting a statistically significant interaction was crucial, as it would indicate that the trajectories of changes in penalty kick performance, and eye-tracking data (fixation duration and fixation count) before and after the brief mindfulness intervention differed depending on the group. Finally, the effects of the brief mindfulness intervention under different stress conditions were compared using independent samples t-tests. All data analyses were conducted using SPSS version 20.0 (IBM). The criteria regarding effect size of Cohen [27]: partialη^2^ (0.04, small effect size; 0.25, medium effect size; and 0.64, large effect size). Diagrams of the results were generated using GraphPad Prism 8.

The eye-movement data analysis was primarily conducted using ErgoLAB 3.0 software, including the categorization of fixation counts and the division of Areas of Interest (AOIs). Based on the players’ gaze habits, four AOIs were defined: the left side of the goal (columns 1 and 2), the center of the goal (columns 3–5), the right side of the goal (columns 6 and 7), and the football. The data that support the findings of this study are available from the corresponding author upon reasonable request.

## Results

### Effects of brief mindfulness training under no-stress conditions

The Shapiro-Wilk (S-W) test confirmed that the data from all groups followed a normal distribution. Additionally, the sphericity test results, with Mauchly’s W equal to 1 and *p* > 0.05, indicated that the data met the sphericity assumption. Repeated measures ANOVA, based on the main effects of time, group, and the interaction between time and group, revealed significant main effects of time and significant time × group interactions for fixation count, and fixation duration (Table 1). Further simple effect and multiple comparison analyses were conducted on these variables.

**Table 1:** Mean, Standard Deviation, Main Effects of Time, Main Effects of Group, and Time × Group Interactions Before and After the Intervention Under No-Stress Conditions(*M ± SD*).

Variables	MM		MW		time			group			time*group		
	pre	post	pre	post	F	η_p_^2^	*p*	F	η_p_^2^	*p*	F	η_p_^2^	*p*
PKP	77.85 ± 10.87	79.35 ± 11.34	76.45 ± 7.26	78.10 ± 8.08	2.85	.07	.10	0.21	.00	.65	0.00	.00	.94
FC	70.35 ± 21.67	85.05 ± 21.51	70.60 ± 18.41	69.10 ± 15.58	9.45^**^	.20	.00	1.85	.05	.18	14.23^**^	.27	.001
FD	0.48 ± 0.07	0.64 ± 0.14^**^	0.51 ± 0.95	0.53 ± 0.15	17.52^**^	.32	.00	2.12	.05	.87	11.44^**^	.91	.86

MM, mindfulness meditation; MW, mind wandering; PKP, Penalty Kick Performance; FC, Fixation Count; FD, Fixation Duration; **, *p* < 0.01.

For penalty scores, A2 (Group: mindfulness vs. control) × 2 (Time: pre vs. post) mixed ANOVA revealed that no significant main effect of group: F(1, 38)= 0.21, *p* = .65, η_p_² = .01; No significant main effect of time: F(1, 38) = 2.85, *p* = .10, η_p_² = .07; No significant interaction effect: F(1, 38) = 0.00, *p* = .94, η_p_² = .00. Neither group showed significant changes in penalty kick scores from pre-test to post-test (*p* > .05).

For fixation count, the ANOVA showed that a significant main effect of time: F(1, 38) = 9.45, *p *< .01, η_p_² = .20; No significant main effect of group: F(1, 38) = 1.85, *p* = .18, η_p_² = .05; A significant interaction between group and time: F(1, 38) = 14.23, *p* < .001, η_p_² = .27; Post-hoc analysis revealed that the mindfulness group significantly increased fixation count from 70.35 ± 21.67 to 85.05 ± 21.51 (*p *< .001), whereas the control group showed no significant change (*p* > .05).

For fixation duration, the ANOVA revealed that a significant main effect of time: F(1, 38) = 17.52, *p* < .001, η_p_² = .32; No significant main effect of group: F(1, 38) = 2.12, *p* = .15, η_p_² = .05; A significant interaction between group and time: F(1, 38) = 11.44, *p *< .001, η_p_² = .91; Post-hoc comparisons showed that the mindfulness group significantly increased fixation duration from 0.48 ± 0.07 s to 0.64 ± 0.14 s (*p* < .001), while the control group showed no significant change (*p* > .05).

## Discussion

In the non-pressure condition, brief mindfulness training significantly increased fixation count and fixation duration, supporting hypothesis 1 and hypothesis 2, indicating enhanced visual attention to task-relevant areas. In contrast, the quiet sitting group (MW group) showed no significant changes in these measures. These findings are consistent with previous research in other sports such as synchronized swimming, snowboarding, and shooting, where brief mindfulness interventions have been shown to improve athletes’ attentional focus and visual search efficiency [16,28,29]. The core of mindfulness training lies in cultivating focused attention and present-moment awareness [11]. By guiding individuals to concentrate on their breathing, bodily sensations, or other present experiences, mindfulness training helps reduce attention to irrelevant thoughts [30]. This process not only enhances mindfulness levels but also improves attention control during tasks, as reflected in the increased fixation count and fixation duration. Furthermore, the increase in fixation measures indicates the positive impact of mindfulness training on participants’ visual attention allocation. Mindfulness training helps individuals maintain more stable and focused attention while performing tasks, rather than frequently shifting focus [10].

The study also found that mindfulness training did not significantly affect penalty kick performance, which fails to support hypothesis 3. This finding aligns with Kaufman [31] et al.‘s research, which also did not observe direct improvements in athletic performance through mindfulness training. This suggests that while brief mindfulness training can positively influence cognitive processes such as attention, its direct impact on task performance may be limited. The duration and frequency of mindfulness training could be crucial factors affecting the results. Mindfulness training typically requires sustained practice over a longer period to produce significant effects in complex sports tasks [32]. However, the brief duration of mindfulness training in this study might not have been sufficient to produce notable effects in a complex skill task like penalty kicks. Additionally, the complexity of penalty kicks itself is an important factor. Penalty kicks not only involve fine motor control but also require psychological readiness and tactical decision-making. Brief mindfulness training may primarily enhance participants’ mental awareness and emotional regulation rather than directly improve such highly automated motor skills [33].

Moreover, although mindfulness training is generally believed to indirectly improve athletic performance by enhancing focus and emotional regulation, its effects may be more evident in training-related concentration or mental state management during competitions rather than in the specific task of penalty kicks [28]. This could be due to the inherent complexity of penalty kicks, which require fine motor skills, psychological readiness, and tactical decision-making, all of which may not be fully addressed by short-term mindfulness interventions.

Although Study 1 did not observe a direct improvement in athletic performance through mindfulness training, its known benefits in reducing anxiety and increasing focus might be more prominent under stress or high-pressure conditions. Therefore, Study 2 will explore the effects of mindfulness training in a pressured environment.

## Study 2: Mindfulness training affect attention and penalty kick performance—Stress condition

### Participants

The participants in Study 2 were recruited using the same criteria as in Study 1. A total of 42 male university football players, all right-footed, with at least three years of football experience, no prior mindfulness training, and no major life events in the past three months, were enrolled. Participation was voluntary, and the study maintained a single-blind design, where participants were unaware of the study’s purpose. Participants were required to have normal or corrected-to-normal vision (e.g., using glasses or contact lenses if needed) and no history of visual impairments such as strabismus, amblyopia, or uncorrected refractive errors that could interfere with eye-tracking calibration and data quality. Participants were recruited through team briefings and informational posters within the university football program. Participation in the study was voluntary, and participants were informed that the research aimed to investigate factors affecting attention and football performance, without disclosing the specific focus on mindfulness training to reduce expectancy effects. Cluster randomization was again used to assign participants to either the control or experimental group. Two participants did not complete the experiment and were excluded from the analysis, resulting in a final sample of 40 participants. The remaining 40 eligible participants were randomly assigned to the MM group (n = 20) or the MW group (n = 20). The average age of the participants was 22.1 ± 2.73 years old. All participants were male, right-footed university football players, experience over a 3–5 year period.

### Procedures

During the preparation phase, each participant was tested individually. After receiving an explanation of the tasks and signing informed consent, participants were fitted with an eye-tracking device and familiarized with its use. They then completed a penalty kick test, and the Mental Readiness Form-3 (MFR-3). Participants in the MM group engaged in 15 minutes of brief mindfulness training using the “Yangqi” app with headphones, while MW group participants sat quietly for 15 minutes. The intervention (“Yangqi” app and sat quietly for 15 minutes) in both study 1 and study 2 was the same. During the penalty kick phase, participants performed 10 penalty kicks, and required to kick within 3 seconds after placing the ball on the penalty spot.

To simulate performance pressure in Study 2, we implemented a multi-faceted pressure manipulation combining time constraint, social evaluation, and monetary incentives, both groups were exposed to pressure conditions. Specifically, participants were: (1) Informed that their performance would be video-recorded and could potentially be shown in class for peer evaluation, increasing social-evaluative threat; (2) Offered monetary rewards, with cash of 200 RMB awarded to the top three performers based on penalty kick accuracy; (3)Required to kick within 3 seconds after placing the ball on the penalty spot, creating a time-pressured execution demand designed to limit preparation and increase decision-making stress. These combined elements were designed to replicate common sources of competitive pressure found in real sports scenarios, such as limited decision time, public scrutiny, and performance-contingent rewards.

## Measurements

### Penalty kick experiment

Used in Study 2 were the same as those in Study 1.

### Eye-tracking hardware

Used in Study 2 were the same as those in Study 1.

### Mental readiness form-3

The Mental Readiness Form-3 (MFR-3) was developed by Jones [34] and has been widely used in sport psychology research. It was translated into Chinese by Liao [35], who also validated its reliability and validity. The MFR-3 measures participants’ anxiety levels and is used for stress manipulation checks. The scale is designed as an 11-point rating system and includes three items that assess cognitive state anxiety, somatic anxiety, and state self-confidence. These three dimensions are measured on scales ranging from “not worried to worried,” “not tense to tense,” and “not confident to confident,” respectively. The state self-confidence dimension is scored in reverse.

## Results

### Stress manipulation check

The effectiveness of the stress condition in the experimental setup was validated using the Mental Readiness Form-3 (MRF-3). The results indicated significant differences in cognitive state anxiety (t = −10.07, *p* = .000 < 0.01) and somatic anxiety (t = −5.45, *p* = .000 < 0.01) between the two conditions (Table 2). These findings confirm that the stress condition implemented in the experiment was effective in successfully inducing different levels of anxiety among the participants.

**Table 2:** Descriptive Statistics of Anxiety (*M ± SD*).

	Under Stress Conditions	No-Stress Conditions
cognitive state anxiety	5.08 ± 2.15	2.58 ± 1.59
somatic anxiety	5.67 ± 2.84	3.17 ± 2.18

### Effects of brief mindfulness training under stress conditions

The Shapiro-Wilk (S-W) test confirmed that the data from all groups followed a normal distribution. Additionally, the sphericity test results, with Mauchly’s W equal to 1 and *p* > 0.05, indicated that the data met the sphericity assumption. Repeated measures ANOVA, based on the main effects of time, group, and the interaction between time and group, revealed significant main effects of time and significant time × group interactions for mindfulness levels, fixation count, and fixation duration (see Table 3). Further simple effect and multiple comparison analyses were conducted on these variables.

**Table 3:** Means, Standard Deviations, Main Effects of Time, Main Effects of Group, and Time × Group Interactions Before and After the Intervention Under Stress Conditions (*M ± SD*).

Variables	MM		MW		time			group			interaction		
	pre	post	pre	post	F	η_p_^2^	*p*	F	η_p_^2^	*p*	F	η_p_^2^	*p*
PKP	62.75 ± 12.58	72.45 ± 11.39	63.75 ± 63.75	68 ± 11.31	8.07^**^	.18	.007	0.28	.01	.00	1.23	0.03	.00
FC	58.90 ± 25.28	77.20 ± 29.23	53.25 ± 16.27	53.95 ± 21.08	17.87^**^	.32	.06	4.17^*^	.10	.048	15.33^**^	0.29	.007
FD	0.54 ± 0.12	0.7 ± 0.22^**^	0.53 ± 0.11	0.56 ± 0.15	24.49^**^	.39	.27	2.62	.07	.00	10.15^**^	0.21	.08

MM, mindfulness meditation; MW, mind wandering; PKP, Penalty Kick Performance; FC, Fixation Count; FD, Fixation Duration **, *p* < 0.01.

For penalty scores, A2 (Group: mindfulness vs. control) × 2 (Time: pre vs. post) mixed ANOVA showed that no significant main effect of group: F(1, 38) = 0.28, *p* = .062, η_p_² = .01; Significant main effect of time: F(1, 38) = 8.07, *p* = .007, η_p_² = .18; No significant interaction: F(1, 38) = 1.23, *p* = .27, η_p_² = .03. Simple effects analysis revealed no significant changes in penalty kick performance from pre-test to post-test in either the mindfulness (*p* = .08 > .05) or quiet sitting group (*p* = .229 > .05).

For fixation count, The ANOVA revealed that significant main effect of group: F(1, 38) = 4.17, *p* = .04, η_p_² = .10; Significant main effect of time: F(1, 38) = 17.87, *p* < .001, η_p_² = .32; Significant interaction effect: F(1, 38) = 15.33, *p* < .001, η_p_² = .29. Post-hoc comparisons indicated that the mindfulness group significantly increased fixation count from 58.90 ± 25.28 to 77.20 ± 29.23 (*p *< .001), while the control group showed no significant change.

For fixation duration, The ANOVA showed that there is no significant main effect of group: F(1, 38) = 2.62, *p* = .114, η_p_² = .07; Significant main effect of time: F(1, 38) = 24.49, *p* < .001, η_p_² = .39; Significant interaction effect: F(1, 38) = 10.15, *p* = .003, η_p_² = .21. These results indicate that mindfulness training significantly improved fixation duration from pre- to post-test.

### Effects of brief mindfulness training under different stress conditions

**Fig 3:**
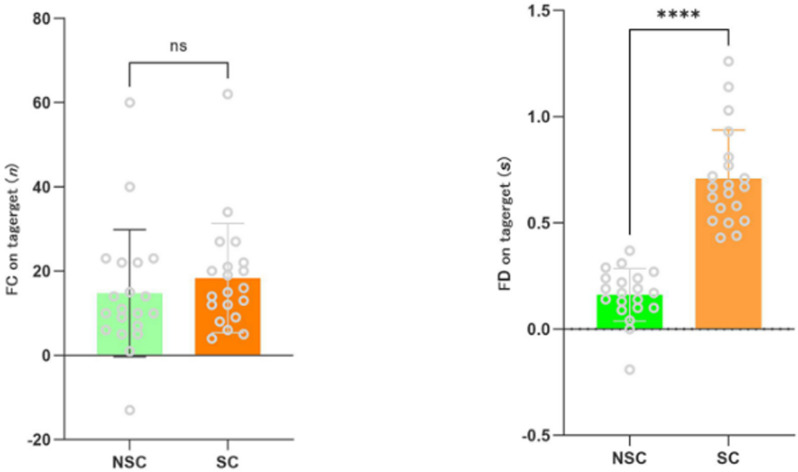
Differences in Fixation Count, and Fixation Duration Within the Mindfulness Group Across Different Stress Conditions. FC, Fixation Count; FD, Fixation Duration; NSC, Non-Stress Condition; SC, Stress Condition; ****,*p* < 0.001.

To further explore the effects of brief mindfulness training under different stress conditions, this study compared the differences in fixation count, and fixation duration within the mindfulness group across varying stress conditions. The primary analysis methods included independent samples t-tests and effect size comparisons. The results indicated that there were no significant differences in the impact of different stress conditions on fixation count (t = 0.31, *p* = .75; t = 0.8, *p* = .42) (see Fig 3). However, there was a significant difference in the impact of different stress conditions on fixation duration (t = 9.49, *p* < .00) (see Fig 3).

## Discussion

The results from Study 2 revealed that brief mindfulness training significantly improved both fixation count and fixation duration under pressure, while no such improvements were observed in the control (quiet sitting) group. These findings support hypotheses 1 and 2, which predicted that mindfulness training would lead to longer fixation durations and higher fixation counts under both non-pressure and pressure conditions. This replication across Study 1 (non-pressure) and Study 2 (pressure) suggests that brief mindfulness interventions can enhance visual attentional processes—such as visual search efficiency and sustained focus—regardless of pressure levels. Specifically, athletes showed improvements in fixation behavior, indicating enhanced task-specific attentional control.

While these improvements in attentional control, as evidenced by increased fixation duration and count, are notable, penalty kick performance did not show a corresponding significant enhancement, failing to support hypothesis 3. This highlights the complexity of translating attentional control improvements into actual performance—especially in the context of short-term interventions. Additionally, this outcome may be partly explained by findings from Norouzi [36], who demonstrated that psychological pressure can impair fine motor skill acquisition by suppressing alpha brain wave activity and disrupting attentional regulation. These physiological disruptions under pressure may override the benefits of short-term psychological interventions, particularly in motor tasks that depend on procedural memory and neuromuscular coordination. Our findings align with this view and suggest that while mindfulness may improve cognitive control, its translation into motor execution under pressure may require either prolonged intervention or concurrent physical training. Moreover, although the sitting group did not show significant improvements in this study, it is worth noting that sitting itself has a certain relaxing effect, which may reduce stress and anxiety to some extent, thereby narrowing the difference between the two groups [9].

This study further explored the effects of brief mindfulness training on participants’ fixation count, and fixation duration under different pressure conditions. The results indicated no significant differences in fixation count between the pressure and non-pressure conditions. However, there was a significant difference in fixation duration, supporting hypothesis 4,and this result is corroborated by some studies [26,37]. Zeidan [12] suggested that brief mindfulness meditation can enhance cognitive functioning and reduce experimentally induced pain perception, especially under pressure conditions, where mindfulness meditation has shown notable effects.

The lack of significant differences in mindfulness levels and fixation count across pressure conditions suggests that, although changes in pressure may affect emotional states and cognitive load, the impact of brief mindfulness training on these variables appears stable and unaffected by pressure. This finding aligns with previous research, which demonstrates that mindfulness training can enhance self-awareness and emotional regulation, maintaining relatively consistent performance in mindfulness levels and visual attention (measured by fixation count) across both high- and low-pressure environments [12,32].

However, for fixation duration, the results indicated significant differences across pressure conditions, suggesting that pressure does indeed affect participants’ visual attention allocation. Under pressure, mindfulness training appeared to have a more pronounced impact on fixation duration, indicating that it helps participants maintain prolonged focus on target areas when facing complex and stressful situations [24]. This finding supports the “Quiet Eye” theory, which proposes that longer fixation durations under high-pressure conditions are often associated with better performance. Mindfulness training may enhance this effect by helping individuals maintain more stable and focused attention [25].

While the present findings are based on controlled laboratory conditions, they have meaningful implications for real-world sports training. Brief mindfulness interventions, such as the 15-minute session tested in this study, offer a time-efficient and accessible strategy for athletes and coaches seeking to enhance attentional control without requiring long-term commitments. Such interventions could be integrated into pre-competition routines to help athletes optimize visual focus, particularly in sports involving self-paced, precision-based tasks like football penalty kicks, basketball free throws, or golf putting [15,24]. Additionally, given the low cost and minimal equipment requirements, this approach is practical for a variety of athletic levels—from youth to elite sports settings [27]. However, to maximize the real-world impact, coaches and practitioners should consider combining mindfulness training with skill-specific physical practice and longer-term mental skills programs. Doing so may better support the transfer of cognitive gains into consistent performance improvements under competitive pressure [12]. Moreover, future field-based studies are needed to validate these findings in authentic competition settings, where dynamic environmental factors and audience effects may further influence attentional and performance outcomes.

In summary, while brief mindfulness training effectively improves cognitive aspects of attention, translating these gains into measurable performance improvements may require longer or more integrated interventions that combine both mental and physical training components.

### Limitations and directions for future studies

Despite providing new insights into the effects of brief mindfulness training under different stress conditions, this study has several limitations. First, the sample size was relatively small, which may limit the external validity of the results. Second, the study only included male university football players, and the results may not be directly generalizable to other populations or fully reflect the performance of higher-level athletes. Moreover, although we made efforts to simulate competition scenarios, there remain significant differences between the laboratory environment and real-world competition settings. The stress condition simulation used in this study may not fully replicate the complex situations and psychological pressures of actual competitions, potentially affecting the external validity of the study results. Finally, one limitation of this study is the use of quiet sitting as a control condition. While it was intended to serve as a non-structured, passive baseline, it does not fully control for expectation effects or the time spent engaging in a structured activity. Although quiet sitting did not involve cognitive tasks, it may have still influenced the participants’ psychological state in ways that could affect attentional control and performance.

Future research could replicate this study with larger and more diverse samples to verify the generalizability of the results. Combining physiological measurements and long-term follow-up studies would help gain a more comprehensive understanding of the long-term impact of mindfulness training on athletic performance and mental health in different contexts.

## Conclusion

This study explored the effects of brief mindfulness training on attentional control and penalty kick performance among university football players under different stress conditions. The results demonstrated that brief mindfulness training significantly improved participants’task-specific attentional control—as evidenced by increased fixation count and fixation duration—during the execution of penalty kicks. These improvements were consistent across both non-stress and pressure conditions, indicating that mindfulness helped athletes maintain greater visual focus and gaze stability in the face of psychological demands. However, the intervention did not produce a direct enhancement in penalty kick accuracy, suggesting that while cognitive attention mechanisms were enhanced, further training may be necessary to translate these gains into improved motor performance.

## Supporting information

S1 File Data. (XLSX)
