# Correction: How does mindfulness training affect attention and penalty kick performance in university football player

**DOI:** 10.1371/journal.pone.0343309

**Published:** 2026-02-17

**Authors:** Jiaqi Wu, Lixin Ai

In Table 3, there are typographical errors in multiple p-values. Please see the correct Table 3 here.

Corrections: The following values have been corrected from the original published version due to table formatting errors during manuscript preparation:

- PKP group main effect: *p* = .062 (was.00)
- PKP Time × Group interaction: *p* = .27 (was.00)
- PKP MW pre-intervention: SD = 13.75 (was 63.75)
- FC time main effect: *p* = .001 (was.06)
- FC group main effect: *p* = .04 (was.048)
- FD time main effect: *p* = .001 (was.27)
- FD Time × Group interaction: *p* = .003 (was.08)

**Table 3:** Means, Standard Deviations, Main Effects of Time, Main Effects of Group, and Time×Group Interactions Before and After the Intervention Under Stress Conditions (M ± SD).

Variables	MM		MW		time			group			interaction		
	pre	post	pre	post	F	η_p_^2^	*p*	F	η_p_^2^	*p*	F	η_p_^2^	*p*
PKP	62.75 ± 12.58	72.45 ± 11.39	63.75 ± 13.75	68 ± 11.31	8.07^**^	.18	.007	0.28	.01	.062	1.23	.03	.27
FC	58.90 ± 25.28	77.20 ± 29.23	53.25 ± 16.27	53.95 ± 21.08	17.87^**^	.32	.000	4.17^*^	.10	.04	15.33^**^	.29	.007
FD	0.54 ± 0.12	0.7 ± 0.22^**^	0.53 ± 0.11	0.56 ± 0.15	24.49^**^	.39	.000	2.26	.07	.114	10.15	.21	.003

MM, mindfulness meditation; MW, mind wandering; PKP, Penalty Kick Performance; FC, Fixation Count; FD, Fixation Duration **, *p* < 0.01.

## References

[1] Wu J, Ai L. How does mindfulness training affect attention and penalty kick performance in university football player. PLoS One. 2025;20(6):e0327134. doi: 10.1371/journal.pone.0327134 40561055 PMC12193906

